# A Comparative Evaluation of Antioxidant Activity of Extract and Essential Oil of *Origanum onites* L. In Vivo

**DOI:** 10.3390/molecules28145302

**Published:** 2023-07-10

**Authors:** Asta Kubiliene, Edvinas Munius, Gabriele Songailaite, Indre Kokyte, Juste Baranauskaite, Arunas Liekis, Ilona Sadauskiene

**Affiliations:** 1 Department of Analytical and Toxicological Chemistry, Faculty of Pharmacy, Medical Academy, Lithuanian University of Health Sciences, Sukileliu St. 13, LT-50161 Kaunas, Lithuania; 2 Department of Pharmaceutical Technology, Faculty of Pharmacy, Yeditepe University, Istanbul 34755, Turkey; 3 Neuroscience Institute, Lithuanian University of Health Sciences, Eiveniu St. 4, LT-50161 Kaunas, Lithuania

**Keywords:** *Origanum onites* L., natural products, liposomes, antioxidant activity, mice

## Abstract

In the present study, the effects of *Origanum onites* L. extract and essential oil of *O. onites* L. on the antioxidant status of the liver and brain of mice were investigated. Due to certain disadvantages of essential oils, such as poor solubility, high volatility and sensitivity to UV light and heat, formulation of liposomes with Oregano essentials (OE) was optimized and used in this study. The results demonstrated that the best composition of the lipid carriers and OE were conducted in terms of the polydispersity index (PDI), mean particle size and encapsulation efficiency (EE). For further study the LE4 formulation was used, which contained Lipoid S100 at 45 mg, Lipoid S75 at 45 mg and 90 mg of EO. The administration of *O. onites* L. extract to mice for 21 days significantly decreased the glutathione (GSH) level in the livers and brains of the mice as well as the malondialdehyde (MDA) concentration in the livers. In the brains of the mice, MDA level significantly increased after this extract consumption. Whereas liposomes with OE significantly decreased GSH concentration in the mouse brain and MDA concentration in the mouse liver, there was an increased (*p* > 0.05) GSH level in the liver and MDA concentration in the brain of mice compared with the control group. It was found that both *O. onites*. ethanolic extract as well as liposomes with OE of this plant material affect the antioxidant status in the livers and brains of mice.

## 1. Introduction

Oxidation reactions are very usual in cells, although they can produce reactive oxygen species (ROS) and free radicals [1]. Oxygen free radicals are products of the normal cellular metabolism and participate as signaling molecules in the regulation of physiological functions and in the redox-regulatory mechanisms of cells in order to protect cells against oxidative stress [2]. However, excessive production of free radicals causes oxidative damage to biomolecules (DNA, lipids, proteins) and is associated with the development of different pathological conditions of the body [2]. The harmful effect of free radicals is termed oxidative stress. Inhibition of oxidative stress can block (suppress) the damage or death of neuronal cells, thus much attention to substances with antioxidant properties is paid. Antioxidants inhibit the oxidation of biomolecules, prevent cell damage and protect the body from the effects of free radicals [3]. As some synthetic antioxidants, such as 2,6-di-tert-butyl-4-methylphenol and 2,4-di-tert-butyl-phenol which are widely used in various commercial products, may cause hepatic toxicity, have endocrine disrupting effects or even be carcinogenic [4], the greatest attention is paid to medicinal plants known or researched to have antioxidant or neuroprotective effects. One such plant is Oregano, used by people for thousands of years.

Oregano is one of the most cultivated aromatic plants in the world. The genus *Origanum* L. (Lamiaceae) consists of 43 species and 15 hybrids [5]. All Origanum species are rich in volatile oils and more than one hundred nonvolatile compounds have already been identified in this plant, which include flavonoids, depsides and origanosides [6]. This abundance of biologically active compounds leads to the different biological properties of Origanum such as antioxidant, antimicrobial, antifungal, antitumor, cytotoxic activity, as well as anti-inflammatory, antispasmodic, antitussive and expectorant properties [1,5,7,8]. Therefore, it is widely use in the treatment of various diseases, such as rheumatism, muscle pains, indigestion, diarrhea, headache and asthma [7]. Medicinal properties are attributed to the antioxidant activity of the essential oil and soluble phenolic fractions [1]. However, there are no consistent studies demonstrating what substances determine the antioxidant activity of Oregano. Some studies attribute the antioxidant activity to the presence of rosmarinic acid [9,10] or to phenolic compounds in the extracts [1,11,12]. However, the antioxidant properties of Oregano are most often attributed to the main components of its essential oil—carvacrol and thymol [8,13]. While *Origanum vulgare* L. is the most commonly studied, our previous study showed the largest amount of carvacrol in *Origanum onites* L. [9,14]. Although the essential oils are primary responsibility for the properties of these plants [1], significant attention is also paid to extract research.

However, essential oils have some disadvantages such as poor solubility, high volatility and sensitivity to UV light and heat and this limits the industrial application of essential oils [15]. It is reported that encapsulation of biologically active compounds is an effective way to increase their stability. One of the widely used methods of nanoencapsulation is liposome technology. Liposomes as a carrier system of essential oils can improve the stability, solubility and bioavailability of bioactive compounds. Liposomes depict spherical bilayer membranes, which are formed by combining one or more various amphipathic phospholipids, yielding nanovesicles (i.e., nanoliposomes) with an aqueous inner core, a hydrophilic inner and outer phosphate surface layer and a hydrophobic lipid bilayer. It has been studied that liposomes as carriers can not only ensure compliance of the physicochemical properties with the proposed stability properties of the active compounds, but also increase the absorption of active substances, prolonging the release of the drug [16].

The main components of the essential oil, in most cases, are carvacrol and thymol [7] while a predominant compound of Oregano extract is rosmarinic acid [17]. However, many monoterpene hydrocarbons and phenolic compounds were also identified in the ethanolic extract, one of the components of which is carvacrol [9,12]. Therefore, both, essential oils and extracts from aerial parts of the plants are used widely because the combination of the compounds present in them seems to be responsible of the activity of these plants, all acting synergistically [1]. In addition, although the essential oil and extract of Oregano have different compositions of biologically active compounds, both have antioxidant properties. Oregano extracts and essential oils are mainly studied in vitro [18], while data on their antioxidant role in vivo are limited [10].

The liver is the main organ exposed to ROS. Parenchymal cells are the first to be affected by oxidative stress. Their mitochondria, microsomes and peroxisomes can produce ROS, and regulate the peroxisome proliferator-activated receptor, which is mainly related to the expression of hepatic fatty acid oxidation genes. Hepatic stellate cells and endothelial cells are the most vulnerable and sensitive to the effects of ROS. Furthermore, oxidative stress not only causes liver damage by causing changes in lipids, proteins and DNA, but also disrupts normal biological functions: gene transcription, protein expression, cell apoptosis [19].

The brain is particularly vulnerable to oxidative stress due to high oxygen utilization. During brain injury such as ischemia, reperfusion and stroke, mitochondrial levels of superoxide anions and ROS are increased [20]. ROS cause DNA damage, lipid peroxidation and protein oxidation in nerve cells, leading to abnormal nerve growth and differentiation. Therefore, it is important that the antioxidant system protects the brain from ROS damage.

Consequently, the aim of this study was to determine the antioxidant activity of *O. onites* in vivo and to evaluate the effect of *O. onites* extract and liposomes with OE on oxidative stress markers (the concentrations of glutathione (GSH) and malondialdehyde (MDA)) in mice organs (the liver and brain). The obtained data allow us to compare the effects of these preparations on the antioxidant system in the body.

## 2. Results

### 2.1. Evaluation of Biologically Active Compounds in O. onites L. Extract and Essential Oil

**Figure 1:**
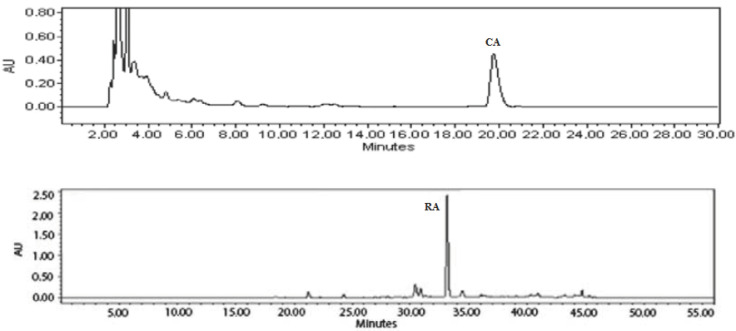
Chromatogram of extract of *O. onites* L. where CA—carvacrol, RA—rosmarinic acid.

Antioxidant activity of Oregano is associated with its constituents rosmarinic acid (RA) and carvacrol (CA). After analyzing the prepared ethanolic *O. onites* extract, it was found that the largest parts in the extract were RA and CA, in compliance with literature data (Figure 1). The highest extracted RA and CA amounts from hydroalcoholic extract were found to be 14.8 µg/g and 22.6 µg/g, respectively.

**Figure 2:**
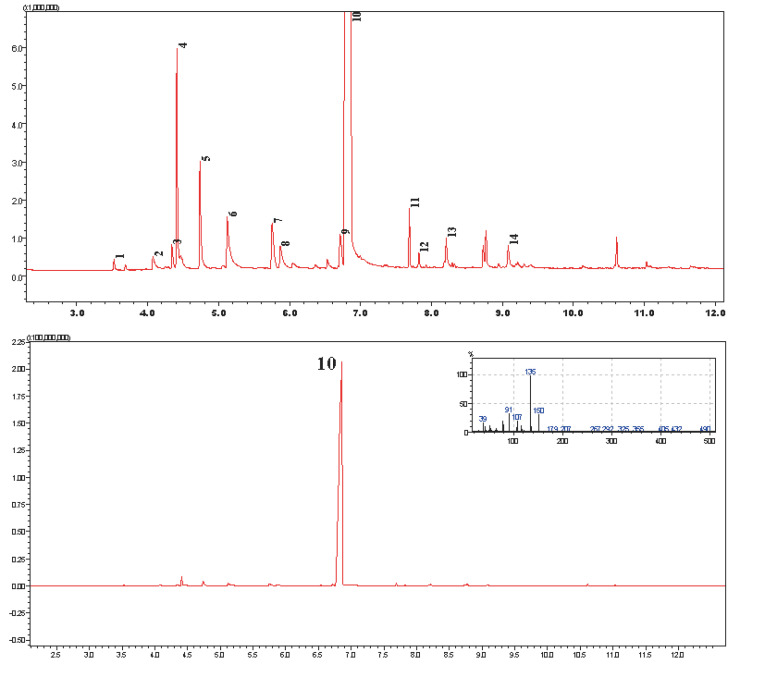
Chromatogram of essential oil of *O. onites* L., where No. 10 is CA. (for information regarding other compounds please see Table 1).

After the essential oil was distilled from the raw material of *O. onites*, it was analyzed to evaluate the compounds it contained and over 14 compounds were identified (Figure 2).

Analysis of the essential oil revealed a large amount of monoterpenes (97.3%), the majority (95.04%) of which was CA, as stated in the literature. Mainly oxygenated-type monoterpenes (96.8%) were identified. Percentage composition of all identified compounds is shown in Table 1.

The concentrations of CA and thymol in the essential oil determined during the quantitative study were 1.35 mg/mL and 0.03 mg/mL, respectively.

The analysis of the extract and essential oil substantiates the presence of the main components of Oregano in the tested preparations. In addition, although the composition of biologically active compounds varies, CA predominates both in the extract and OE.

### 2.2. Characterization of Liposome Formulation

The unloaded liposomes were produced by the Bangham method. The compositions of the prepared unloaded liposome formulations are presented in Table 4. In order to determine the most suitable matrix of the unloaded liposome formulation the following parameters were measured: mean particle size (nm), PDI and zeta potential (mV). The obtained results are presented in Table 2. The mean particle size of unloaded liposome formulations varied between 63.53 ± 1.13 nm and 164.70 ± 0.95 nm. The smallest size (63.53 ± 1.13 nm) was observed in the L5 formulation and the highest (164.70 ± 0.95 nm) in the L2 formulation. Moreover, the zeta potential of the formulations ranged from −7.78 ± 8.07 mV to −29.94 ± 1.80 mV. According to the obtained results, the formulations were stable. The PDI parameter indicates the monodispersity of the liposome formulation particles. It was found that the PDI of the samples ranged from 0.26 ± 0.01 to 0.33 ± 0.03. The produced formulations had a homogeneous particle size distribution. According to the results, for further study the L2 and L3 formulations were selected.

**Figure 3:**
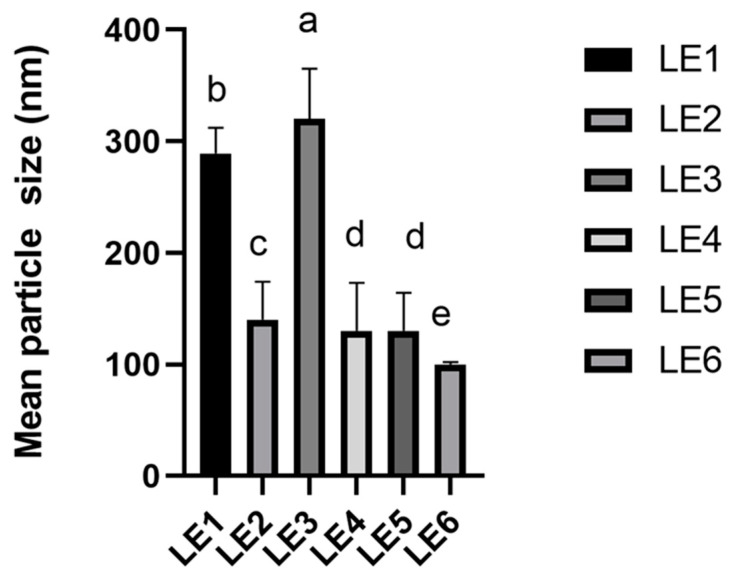
The mean particle size of the OE loaded liposome formulations. Results are expressed as mean ± standard deviation, *n* = 6. Different letters in each column denote a statistical difference at *p* ≤ 0.05. The composition of the liposome formulation is shown in Table 5.

The compositions of the OE loaded liposomes are shown in Table 5. In order to choose the most suitable lipid nanocarriers with encapsulated EO, the physicomechanical properties (the mean particles size; zeta potential and PDI) of the formulations were evaluated. The particle size of loaded liposomes varied from 103.90 ± 4.86 nm to 323.7 ± 26.60 nm (Figure 3). The obtained results showed that the largest particles size (323.7 ± 26.60 nm) was obtained when Lipoid S100 to OE was 1:5 and the lowest when the ratio of phospholipid mixture (Lipoid S75 and Lipoid S100) and OE was 1:5.

**Figure 4:**
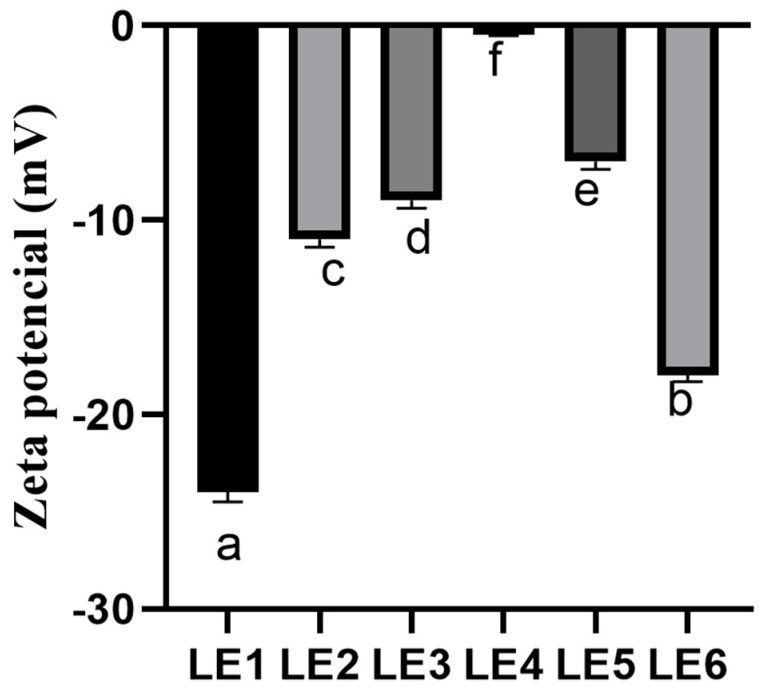
The zeta potential of the OE loaded liposome formulations. Results are expressed as mean ± standard deviation, *n* = 6. Different letters in each column denote a statistical difference at *p* ≤ 0.05. The composition of the liposome formulation is shown in Table 5.

The zeta potential is one of the most important parameters in determining the stability of lipid nanocarriers. The zeta potentials of the produced OE loaded liposome formulations are presented in Figure 4. The obtained results showed that the zeta potential was below −30 mV in all formulations. The results confirm that the phospholipid mixtures of Lipoid S75 and Lipoid S100 are suitable to produce stable nanoparticles.

**Figure 5:**
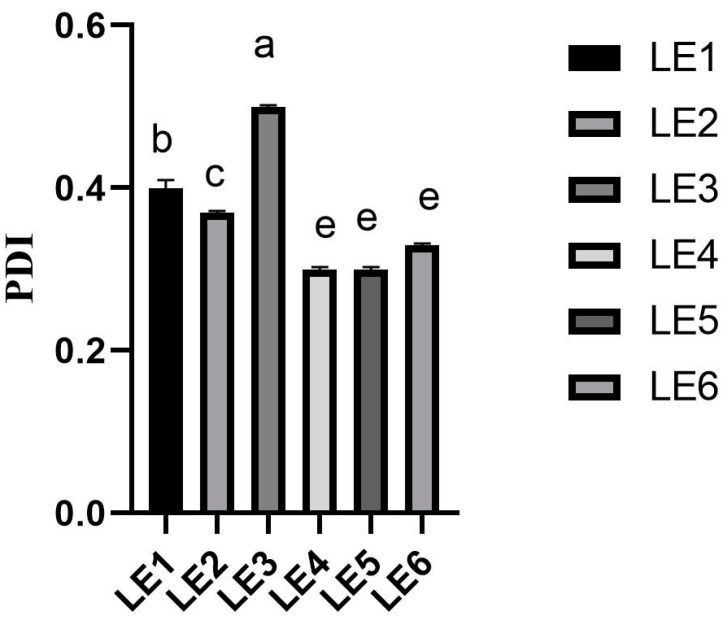
The PDI of the OE loaded liposome formulations. Results are expressed as mean ± standard deviation, *n* = 6. Different letters in each column denote a statistical difference at *p* ≤ 0.05. The composition of the liposome formulation is shown in Table 5.

The PDI of the OE loaded liposomes during the study was distributed within the range of 0.29 ± 0.02 and 0.31 ± 0.04 (Figure 5). Moreover, it was found that the LE3 PDI value was greater than 0.3 and this showed that the prepared formulation could be unstable. The instability of the LE3 could be caused by the high amount of OE.

According to physicomechanical properties of the OE loaded liposome formulation, for further study LE4, LE5 and LE6 were chosen.

### 2.3. Encapsulation Studies

**Figure 6:**
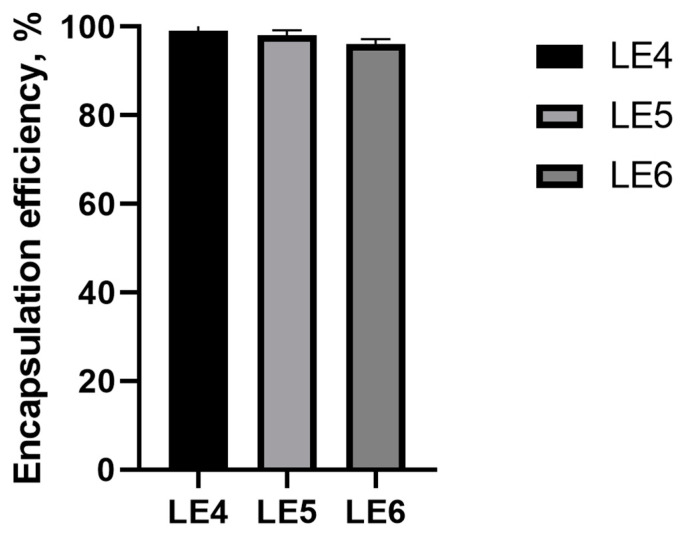
The encapsulation efficiency of prepared LE4, LE5, LE6 formulations. Results are expressed as mean ± standard deviation, *n* = 6. The composition of the liposome formulation is shown in Table 5.

The EE of the CA in the LE4, LE5 and LE6 formulations were evaluated and presented in Figure 6. The results varied between 96.99 ± 1.21% and 99.34 ± 0.51%. The highest EE of CA was observed in LE5 and the lowest in the LE6 formulation. Results showed that lipid concentration affects the encapsulation efficiency of CA.

### 2.4. Stability Studies

The stability of LE4 and LE5 was tested after 1 month at 5 ± 3 °C temperature (Table 3). In all aspects of testing, LE4 and LE5 appeared physically, chemically, mechanically and organoleptically stable. The mean size, zeta potential, PDI and EE of CA were all comparable at the initial time and after 1 month. In the LE4 liposome formulation the mean particle size changed from 127.90 ± 1.81 to 129.70 ± 1.22 nm, the zeta potential from −0.31 ± 0.13 mV to −0.29 ± 0.26 mV and the PDI remained at 0.3. In the LE5 formulation, the same changes in results were observed: the particle size after 1 month was 132.20 ± 2.60 nm, the zeta potential was −7.77 ± 1.31 mV and the PDI, as in the LE4 formulation, (*p* > 0.05). The slight decrease in EE of CA results was observed after one month in the LE4 formulation. In the LE4 formulation, the EE of CA decreased by 2.43%.

### 2.5. Results of MDA

**Figure 7:**
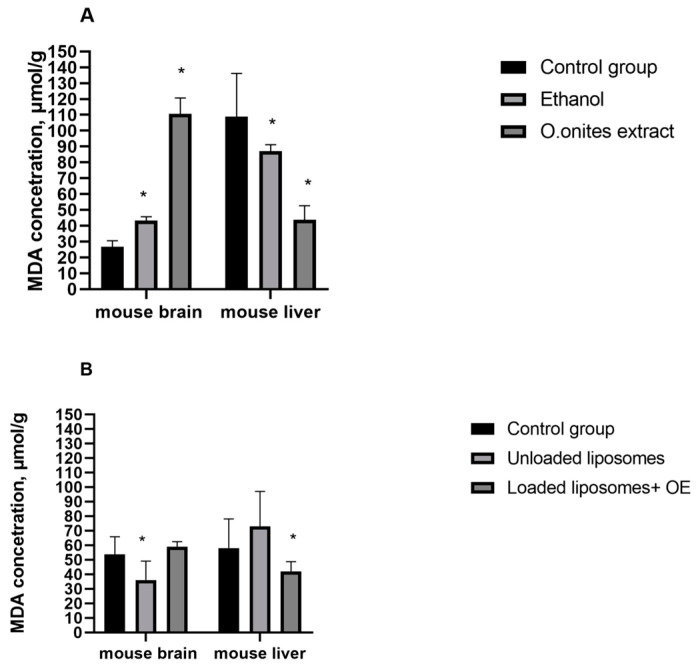
MDA concentration in the mouse liver and brain after *Origanum onites* L. extract (**A**) and loaded liposomes with essential oil of *Origanum onites* L. (**B**), where control group—control 1, ethanol—control 2 group, unloaded liposomes—control 3 group. The mice of control 1 group received a saline solution (*n* = 8, * *p* < 0.05 vs. control 1).

Results on the level of MDA in the liver and brain of control and experimental animals are given in Figure 7A,B. The results revealed that MDA levels were greater in the brain of the mice after supplementation of ethanolic *O. onites* extract (*p* ≤ 0.05) or OE loaded liposomes (*p* > 0.05) compared with that in the vehicle control (control 1) group. Whereas both extract and OE loaded liposomes decreased (*p* ≤ 0.05) MDA levels in the liver of mice compared with the same control 1 group.

We evaluated the MDA concentrations in the brains and livers of experimental animals on ethanol and blank liposome supplementation (control 2 and control 3 group, respectively). MDA concentration in mice brains in control 2 group (after ethanol supplementation) was significantly higher (*p* ≤ 0.05) compared with the control mice (increased 94%), whereas blank liposomes (control 3) decreased (*p* ≤ 0.05) the MDA concentration (33%) compared with the control mice (control 1 group). While, on the contrary, the MDA concentration on ethanol supplementation was significantly lower (20%) compared with the control mice (control 1 group), but blank (unloaded) liposomes increased (*p* > 0.05) the MDA concentration in mice livers.

The amount of MDA in mice brains after use of *O. onites* ethanolic extract was significantly higher compared with control 2 group (Figure 7A). Meanwhile, this concentration in the mice livers was significantly decreased. The same trend was observed in comparison of OE loaded liposomes–loaded liposomes increased MDA concentration significantly in the brains but decreased it in the livers of mice, compared with control 3 group (unloaded liposomes) (Figure 7B).

### 2.6. Results of GSH

**Figure 8:**
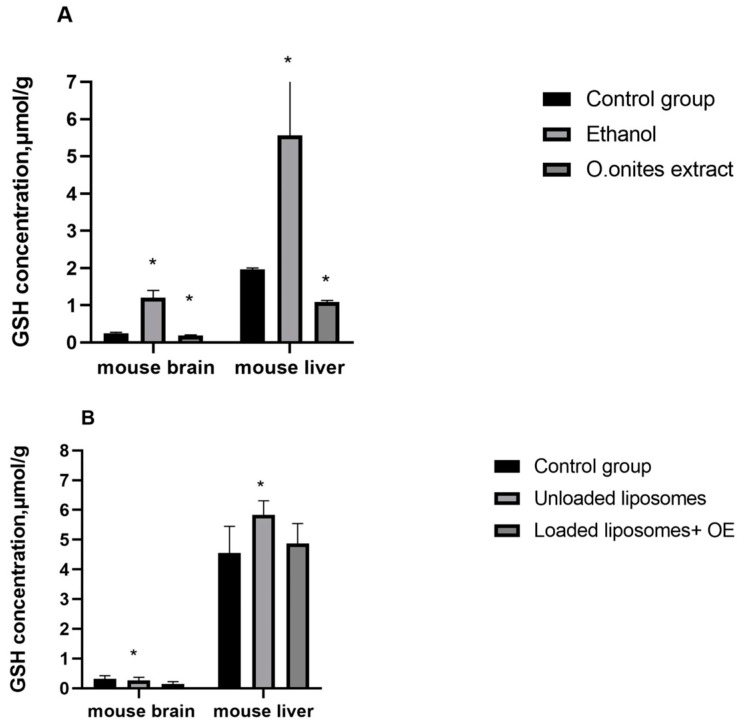
GSH concentration in the mouse liver and brain after *Origanum onites* L. extract (**A**) and loaded liposomes with essential oil of *O. onites* L. (**B**), where control group—control 1, ethanol—control 2 group, unloaded liposomes—control 3 group. The mice of control 1 group received a saline solution (*n* = 8, * *p* < 0.05 vs. control 1).

The concentrations of GSH in the livers and brains of control and experimental mice are given in Figure 8A,B. The obtained results showed that ethanolic *O. onites* extract decreased (*p* ≤ 0.05) GSH level in both the liver and brain, by 24% and 45%, respectively. Whereas liposomes with OE reduced GSH concentration in the brain (*p* ≤ 0.05) it increased in the liver (*p* > 0.05) compared with the control mice (control 1).

We evaluated the GSH concentrations in the livers and brains of mice on ethanol and blank liposome supplementation too. The concentrations of GSH in mice brains and livers that received ethanol (control 2) were significantly higher than that in control 1 group. Meanwhile, in control 3 group (that received unloaded (blank) liposomes)—GSH concentration was 17% lower in the brains (*p* > 0.05) but 28% higher in the livers of the mice (*p* ≤ 0.05) compared with control 1.

GSH levels in both liver and brain were significantly (*p* ≤ 0.05) lower after ethanolic *O. onites* extract consumption compared with the ethanol control group (Figure 8A) as well as after liposomes loaded with OE compared with control 3 group (unloaded liposomes) (Figure 8B).

## 3. Discussion

Liposomes as a nano delivery system were used in this research due to their excellent encapsulation efficiency, biocompatibility and safety [21]. The physicomechanical properties of liposome formulation were measured. The mean particle size of lipid nanocarriers is one of the most important parameters in the evaluation the quality of the produced liposomes. The obtained results showed that the mean particle size of the produced formulations depended on the ratio of phospholipid in the mixture. According to scientific publications, if the size of the liposome is between 70 nm and 200 nm, it is considered stable, it will last longer in the systemic circulation and have a higher probability of reaching the desired target site [22]. Results showed that the mean particle size of the formulations met the requirements. Moreover, the results showed that the increase in mean particle size in the OE loaded formulations was influenced by increasing the amount of essential oil. These results could be explained by the chemical structure of OE, with increasing hydrophobic phase the interaction with the acyl group in the phospholipid increases, so the transfer across the membrane layer may deteriorate [16]. Lastly the homogeneous particle size distribution was obtained of the OE loaded liposome formulation produced by mixture of Lipoid S75 and Lipoid S100 phospholipids [16].

The zeta potential is an important parameter in evaluating the physical stability of lipid nanocarriers. According to the literature, nanocarriers are considered to be stable if the zeta potential of the particles is ≤−30 mV or >30 mV [23]. The produced formulations were stable. Furthermore, physical stability of the formulations was influenced by the phospholipid mixture. Eroğlu and co-authors (2014) in their research used phospholipid Lipoid S100 to form the lipid nanocarriers and the zeta potential of the produced lipid nanocarriers ranged from −28.90 ± 0.80 mV to −24.50 ± 0.60 mV [24]. Moreover, in another study, liposomes were produced with phospholipid Lipoid S75 and the zeta potential reached −12.6 ± 0.6 mV [25]. Summarizing the results of other authors, it can be stated that, in order to produce stable nanoparticles, it is relevant to choose a mixture of Lipoid S75 and Lipoid S100 phospholipids for the formulation of lipid nanocarriers. Lastly, only in the LE3 formulation was the PDI greater than 0.3. The instability of the formulation could be explained by an increased amount of essential oil. Moreover, this instability could be explained by the chemical structure of OE where the active compounds can interact with membrane layer. The obtained results were in agreement with other authors [14].

The PDI value is a significant factor in terms of showing the size distribution of the liposomes and being correlated with the distribution stability. A PDI value of 1.0 specifies a very wide size distribution or the presence of large particles that can precipitate. An optimum PDI value is 0.30 or less, indicating that 66.7% of nanovesicles are the same size [26]. All of the prepared formulations had a homogenous particle size.

In order to evaluate the quality of lipid nanocarriers, it is important to determine the active compound encapsulation efficiency and it should be close to 100%; then, the maximum amount of active compounds is encapsulated in lipid carriers [16]. The obtained results showed that an increased lipid concentration in the formulation could affect the encapsulation efficiency results. The highest encapsulated amount of CA was obtained in the formulation with a mixture ratio of essential oil and phospholipids (Lipoid S75 and Lipoid S100) of 1:5. It has been observed that the encapsulation ability decreases as the lipid concentration decreases in the formulation [16].

A wide spectrum of important classes of active principles was detected in Oregano, such as essential oil (with CA and/or thymol, linalool and p-cymene), polyphenols (flavonoids and phenolic acids), triterpenoids and sterols [10]. Terpenes, mainly CA and thymol, are characteristic and the main active compounds of the essential oil of Oregano species [7] which have the highest antioxidant effect [13]. Studies have demonstrated the antioxidant, neuroprotective, antimicrobial, anti-inflammatory, antiviral, antiproliferative, etc. effects of *Origanum vulgare* L. extract [10]. However, increasing scientific attention is directed towards *O. onites* for its antioxidative, antibacterial, antifungal and insecticidal effects on human health [27] *O. onites* showed strong antioxidant activity in different tests of extracts obtained from several solvents [13].

A total of 134 terpenoids, 6 phenylpropanoids and 26 other components have been identified in the physicochemical studies of essential oil of *O. onites* [13].

The antioxidant activity of *O. vulgare* extract evaluated using FRAP, CUPRAC, inhibition of lipid peroxidation catalyzed by cytochrome c and superoxide (SO) scavenging assays [10]. The obtained results indicated a high antioxidant potential of this extract in vitro, in line with the total polyphenolic content. The possibility of *O. vulgare* extract therapy as well as RA and CA (active compounds of Oregano), to restore the antioxidant enzyme activity and to facilitate lipid peroxidation in vivo was detected [10,28].

The present study evaluated the effects of *O. onites* ethanolic extract and essential oil as well as ethanol and blank liposomes on the oxidative stress markers in the brain and liver cells of mice.

Oxidative stress can cause damage to cell structures and consequently leads to various diseases and ageing [2]. In the case of oxidative stress, ROS form. However, the accumulation of ROS is controlled in vivo by a wide spectrum of non-enzymatic antioxidant systems, such as GSH that scavenges free radicals or act as a GSH peroxidase substrate [28], i.e., it has the main role in coordinating the antioxidant defense processes in the body.

In our previous study, we investigated that the main components of Origanum, CA and RA, reduce the concentration of GSH in the brains and livers of mice. Therefore, in this study we investigated the effect of the whole extract or essential oil of *O. onites* on GSH concentration as well as in the presence and absence of ethanol (control 2) or unloaded (blank) liposomes (control 3). Our results indicated that ethanolic Origanum extract decreased this concentration in the brains as well as in the livers of mice, in agreement with previous studies that assessed the effects of RA (main compound of Origanum extract) and CA l on GSH levels in mice brains and livers [28]. As ethanol increased the GSH concentration in both the liver and brain, this suggests that the decrease of this concentration is influenced by the active compounds of the extract.

Whereas the effects of liposomes with OE also showed a decrease in GSH levels in the brain, in agreement with study of the effect of the main essential oil component—CA—on the GSH level in the brain, it caused a slight increase in the liver. Since the essential oil is practically made up of CA, such results could be caused by the phospholipids contained in the liposomes since the administration of unloaded liposomes also showed an increase in GSH concentration. However, since the diminished GSH level in the liver after administration of liposomes with OE was not statistically significant, this suggests the potential of preparations to act as liver and brain protectants via the antioxidant effect.

MDA level indicates the extent of oxidative stress as it is the end product of lipid peroxidation [28]. We evaluated the changes of MDA concentrations in the livers and brains of mice in the presence and absence of extract or liposomes loaded with OE as well as in the presence and absence of ethanol or blank liposomes. Our results indicated that, after 21 days of supplementation of ethanolic *O. onites* extract, the experimental mice demonstrated significant changes of the concentration of MDA in the liver and brain, compared with control values. Meanwhile, liposomes with OE demonstrated marked changes of MDA only in the livers of mice. However, while MDA concentrations in the brain had a tendency to be elevated, they were significantly decreased in the liver after extract or liposomes with essential oil of *O. onites*. As ethanol increased the MDA level in the brains of mice, this suggests that the solvent of extraction or other minor components present in the extract affect the concentration of MDA. Contrary changes in the MDA concentrations were observed after administration of blank liposomes in the liver and brain compared to control values: the MDA level decreased in the brain while it increased in the liver. Compared to blank liposomes, OE loaded liposomes increase the MDA level in the brain but decrease it in the liver. Results in the liver show that liposomal phospholipids increase MDA levels in the liver but the addition of an essential oil with CA as its main component has the opposite effect. The same effect of CA on MDA concentration in the liver was found in our previous [19] study.

Our results indicated that both the ethanolic extract and liposomes with essential oil of *O. onites* reduce MDA concentrations in the livers of mice and it is in agreement with a previous study of RA and CA [28]. The main components of extract and essential oil—show the ability of studied preparations to reduce oxidative stress. However, results in the brain were controversial. While RA as well as CA decreased MDA concentration in the brain, both Oregano extract and OE loaded liposomes increased levels of this enzyme.

The differences of changes of MDA and GSH levels in experimental mice brains and livers after administration of extract or liposomes with OE allow us to agree with the statement that pro-oxidant and antioxidant activities could be noticed at different doses of phenolic compounds [29].

In general, our results obtained that ethanolic *O. onites* extract as well as OE loaded liposomes after 21 day of the experiment affected concentrations of GSH and MDA. Oregano extract and liposomes with essential oil significantly changed both MDA and GSH concentrations in mice livers and brains. A non-significant % increase of MDA concentration in the brain and GSH in the liver were obtained after administration of OE loaded liposomes. However, our study results showed that these liposomes significantly reduced the concentrations of tested oxidative stress markers, except the MDA level in the brain, where a significant increase was detected compared to administration of blank liposomes.

Administration of ethanol solution, which is the base of the extract, affected the MDA level in the livers and brains of mice as *O. onites* extract did, while the effect on changes in concentration of GSH was the opposite.

The results of the study show that the main effect on the concentration of oxidative stress markers MDA and GSH is due to the main component CA present in the extract and essential oil and RA in the extract. However, extract and essential oil of *O. onites* have different effects on oxidative stress markers in the body. In addition, although it does not allow us to unequivocally state that the *O. onites* extract and OE loaded liposomes can reduce the level of oxidative stress markers in vivo, however, a mild degree of oxidative stress can increase the levels of antioxidant defenses and xenobiotic-metabolizing enzymes, leading to overall cytoprotection. Thus, the pro-oxidative effect can be beneficial in practice [30].

## 4. Materials and Methods

### 4.1. Plant Material

Dried *Origanum onites* L. herb was purchased from “İnanTarım ECO DAB” Turkey. Voucher specimen (number L170711) has been deposited at the Herbarium of the Department of drug technology and social pharmacy, Lithuanian University of Health Sciences, Lithuania according to the requirements of the European Pharmacopoeia (monograph number 01/2011:1880). The test used includes a pharmacognostical investigation of the anatomical and microscopical characteristics and TLC fingerprinting. The dried herb particle size was 125 μm.

### 4.2. Reagents

Lipoid S75, Lipoid S90 and Lipoid S100 were purchased from Lipoid GmbH, Ludwigshafen, Germany.

Methanol (99%) was supplied by Carl Roth GmbH, Karlsruhe, Germany.

Potassium chloride, hydrogen peroxide, phosphoric acid were supplied by Merck, Darmstadt, Germany.

Ethanol (96%) was purchase from Vilnius degtine, Vilnius, Lithuania.

TBA (thiobarbituric acid), tris-HCl (tris-hydrochloride), DTNB (5,5-dithiobis-(2-nitrobenzoic acid) were supplied by Serva, Heidelberg, Germany.

Formic acid was purchased from Fluka Chemie, Buchs, Switzerland.

Purified water was produced using a Millipore water purification system (Merck, Rahway, NJ, USA).

Acetonitrile, chloroform (99.8%), acetic acid (99.8%), thymol (99%) and CA (98%) were supplied by Sigma-Aldrich, Buchs, Switzerland.

Helium (99.999%) was supplied by AGA, Vilnius, Lithuania.

### 4.3. Preparation of Extract

The preparation of extract was done based on the article of Baranauskaite et al. in 2017 [9]. A total of 5 g of dried, powdered plant material was poured into 100 mL of 90% (*v*/*v*) ethanol and extracted in a round bottom flask by heat-reflux extraction performed in a water bath for 4 h at 95 °C [9]. The extract was passed through a 0.22 μm membrane filter. The extract was diluted to 10% ethanol concentration (1:200) before the administration to the mice.

### 4.4. Preparation of Essential Oil

A total of 50 g of plant material with 500 mL of water were placed in a round-bottomed flask. The unit was carried to glycerol batch of 120 °C temperature for 2 h. The vapor mixture of water–oil produced in the flask passes to the condenser, where it was condensed. After condensation, the oil was separated from water by decantation, measured on an analytical scale and kept in a cool, dark place.

### 4.5. Analysis of Extract

The HPLC methodology used for extract analysis was described in our previous study in 2017 [9]. Waters 2695 chromatography system (Waters, Milford, CT, USA) equipped with Waters 996 PDA detector and 250 × 4.6 mm 5-µm ACE C18 column (Advanced Chromatography Technologies, Aberdeen, UK) for investigation of biologically active compounds in extract of *O. onites* L. was used. Different HPLC conditions for determination of the main compounds of the extract (RA and CA) were used.

Determination of RA: the mobile phase consisted of a mixture of methanol (solvent A) and 0.5% acetic acid (*v*/*v*) (solvent B). Gradient elution profile was used as follows: 95% A/5% B–0 min, 40% A/60% B–40 min, 10% A/90% B–41–55 min and 95% A/5% B–56 min. The solvent flow rate was 1 mL/min and the injection volume was 10 μL. The identification was carried out at a wavelength of 329 nm.

Determination of CA: the mobile phase consisted of a mixture of methanol and water (60/40, *v*/*v*). The solvent flow rate was 0.6 mL/min, injection volume was 10 μL. The absorbance was measured at a wavelength of 275 nm.

Quantification of compounds was carried out by the external standard method. Standard stock solutions at a concentration of 1.0 mg/mL were prepared in methanol and diluted in appropriate quantities to obtain a set of corresponding concentration ranges for the study of linearity.

### 4.6. Gas Chromatography Analysis of Oregano Essentials

The initial column temperature was 60 °C, injector temperature 290 °C, amount of injected sample 1 μL. Helium was used as the carrier gas. The temperature was raised gradually: from 70 °C to 250 °C, 20 °C/min. speed, from 250 °C to 300 °C raised at 50 °C/min. speed. One test sample was analyzed for 15 min. Chromatograms analyzed Lab Solution GMSS solution Shimadzu program. OE identification of components was performed by comparison of the retention indices of the peaks with literature values [31] and by GC/MS libraries NIST14, NIST14s, WR10, WR10R Mass spectral program by analyzing mass spectra. The Kovat indices were obtained from the gas chromatograms by comparing with the natural homologous series of hydrocarbons. The identity was further confirmed by matching their mass spectra with those recorded in NIST library search.

Quantifications of CA and thymol were calculated from peak areas using a calibration curve (R^2^ for CA = 0.999751, R^2^ for thymol = 0.9968) according to the external standard method.

### 4.7. Preparation of Unloaded Liposomes

The liposomes were prepared by the Bangham method [32]. The phospholipid mixture was dissolved using 2:1 (*v*/*v*) chloroform/methanol in round-bottomed flasks. The composition of the phospholipid mixture can be seen in Table 4. The organic solvents were removed (20 min, 35 °C) using a rotary evaporator (Schwabach, Germany) and in the last step, solvent residue was removed with N_2_ gas.

### 4.8. Preparation of OE Loaded Liposomes

To prepare the uniform particles by avoiding clustering into unloaded liposomes, OE was incorporated according to the Bangham method [32]. To prepare loaded liposome formulations (LE1, LE2, LE3, LE4, LE5 and LE6) unloaded liposome L2 and L3 composition and desired OE were dissolved into a 2:1 (*v*/*v*) chloroform/methanol solvent mixture in round-bottomed flasks. The organic solvents were removed (20 min, 35 °C) using a rotary evaporator (Schwabach, Germany) and solvent residue was removed with N_2_ gas. The thin film was dispersed into purified water and was homogenized (7k rpm, 8 min) using a Wise Tis HG-15D model homogenizer. The composition of loaded liposomes formulations is shown in Table 5.

### 4.9. Characterization of Particle Size Distribution and Zeta Potential

Differential light scattering (DLS) (Nano ZS 3600, Worcestershire, UK) was used to measure mean particle size and PDI. A low (<0.25) PDI value indicates a homogeneous particle size distribution, while a high value (>0.5) indicates heterogeneity. Measurements of the zeta potential were also obtained by operating the DLS in zeta mode. Electrocuvettes were used to measure the zeta potential.

### 4.10. Encapsulation Efficiency

The encapsulation efficiency (EE) of the liposomes was determined by centrifuging the formulation at 3000 *g*×s rpm for 20 min (Spectra Por, Darmstadt Germany). The amount of active substance in the collected filtrate was analyzed using the GC-MS method, and the amount of drug dissolved in water, if any, was also determined. For this purpose, the formulations were taken into a chambered centrifuge tube containing a membrane with a pore opening of 10 kDa (Merck Millipore, Merck KGaA, Darmstadt, Germany) and centrifuged at 10,000 rpm for 30 min, and the amount of active substance in the separated water phase (filtrate) was determined by GC-MS method (GC–QP2010, Kyoto, Japan) method [16]. The EE was determined by the formula:

(1) $$EE\left(\%\right)=\frac{qp}{qt}\times 100$$

where qp is the amount of the active ingredients in liposomes (mg/mL) and qt is the total amount of active ingredients added to the liposomes (mg/mL).

### 4.11. Evaluation of Stability

To study storage stability, liposomes were stored (4 °C, 1 month) in amber colored glass containers (4 oz.). Parameters were assessed such as particle size, zeta potential, PDI, EE% of CA.

### 4.12. Animal Model

The experiment was carried out on 6–8-week-old white female BALB/c laboratory mice weighing 20–25 g. Tests with animals were performed according to the study protocol and were approved by the Lithuanian State Food and Veterinary Service, License no. G2–203.

The extract and liposomes (10 mL/kg body weight) were administrated intragastrically for 21 days. The mice received feed and drinking water *ad libitum* throughout the experiment and were kept on a 12 h light−dark cycle. The mice were kept in group cages, containing 8 mice each:

- Control 1 group received NaCl 0.9% (the saline solution) for 21 days.
- Control 2 group received 10% ethanol solution.
- Control 3 group received blank liposomes (unloaded liposomes).
- The 4th group received ethanolic *O. onites* extract.
- The 5th group received liposomes with *O. onites* essential oil (loaded liposomes + OE).

MDA and GSH concentrations were examined in all tested groups and compared with the control 1 group. These parameters were also assessed compared to ethanol group (control 2 group) or to blank liposomes group (control 3 group).

The brain and liver homogenates were prepared according to the procedure in Baranauskaite et al. [28]. The brains and livers of the animals were removed, washed and immediately cooled on ice after cervical dislocation. Weighed organs were homogenized with 9 volumes of cold 1.15% KCl solution (relative to organ weight). This results in a 10% homogenate, which was further centrifuged at 15,000× *g* for 15 min.

### 4.13. Evaluation of GSH

GSH concentration was estimated by the method described by Sadauskiene et al. [33]. Weighed mouse liver or brain was homogenized in a 6-fold (relative to tissue weight) volume of 5% trichloroacetic acid solution and further centrifuged at 10,000× *g* for 7 min. GSH concentration was determined after reaction with DTNB (Ellman’s reagent or 5,5′-dithiobis (2-nitrobenzoic acid)). Each sample (3 mL) consisted of 2 mL of 0.6 mM DTNB in 0.2 M sodium phosphate (pH 8.0), 0.2 mL of supernatant fraction and 0.8 mL of 0.2 M phosphate buffer. The formed compound absorbs light with a wavelength of 412 nm. The content of GSH was expressed as μmol/g of wet tissue weight.

### 4.14. Evaluation of MDA

MDA is one of the markers of lipid peroxidation which forms a complex with thiobarbituric acid (TBA) and can be detected by spectrophotometry. MDA content was evaluated in brain and liver samples and the obtained results were expressed in nmol/g of wet tissue weight [33]. For sample preparation, 0.5 mL of liver/brain homogenate, 3 mL of 1% phosphoric acid and 1 mL of 0.6% TBA solution were mixed in a test tube. This mixture was incubated in boiling water bath for 45 min, cooled and mixed with 4 mL of n-butanol. Light absorbance of the supernatant was determined at 535 and 520 nm after separation of the butanol phase by centrifugation [33].

### 4.15. Statistical Analysis

Data were assessed using unpaired Student’s *t*-test and the nonparametric Wilcoxon criterion for dependent samples. Statistical significance was taken as a value of *p* ≤ 0.05 (SPSS version 20.0, SPSS). Data were expressed as the mean ± SEM (standard error of mean) (*n* = 8).

## 5. Conclusions

The used method of preparation of unloaded liposomes and OE loaded liposomes allowed the production of high-quality liposomal systems. The liposome formulations with the best physicomechanical properties were used for further in vivo studies. To conclude, our study revealed that prolonged exposure to ethanolic extract or liposomes with OE has a different effect on oxidative stress markers in the livers and brains of mice. *O. onites* ethanolic extract decreased (*p* < 0.05) the concentrations of GSH in the brains and livers of mice and MDA concentration in the livers of mice but increased (*p* < 0.05) the concentration of MDA in the brain of mice. Whereas liposomes with OE decreased (*p* < 0.05) GSH concentration in the brains and the MDA concentration in the livers of mice but increased the (*p* > 0.05) GSH amount in the brains and MDA in the livers.

## Figures and Tables

**Table 1:** The percentage composition of compounds determined in essential oil of *O. onites* L.

Peak No.	Compound	Composition	Kovat Index (Obtained)	Kovat Index (lit. Value)
1.	α-Pinene	0.06%	935	932
2.	Myrcene	0.16%	989	988
3.	2-Carene	0.17%	998	1001
4.	o-Cymene	1.19%	1037	1022
5.	α-Pinene oxide	0.75%	1083	1099
6.	Linalool	0.63%	1088	1095
7.	Isoborneol	0.55%	1157	1155
8.	Terpinene-4-ol	0.27%	1176	1174
9.	Thymol	0.30%	1272	1289
10.	Carvacrol	95.04%	1277	1298
11.	α-Bisabolene	0.32%	1488	1506
12.	Aromadendrene	0.11%	1490	1439
13.	β-Bisabolene	0.26%	1507	1505
14.	Cadinol	0.18%	1654	1652

**Table 2:** Physicomechanical properties of prepared unloaded liposomes. Results are expressed as mean ± standard deviation, *n* = 5. Different letters in each column denote a statistical difference at *p* ≤ 0.05. The liposome formulation codes are shown in Table 4.

Formulation	Physicomechanical Properties		
	Mean Particle Size (nm)	Zeta Potential (mV)	PDI
L1	70.67 ± 1.90 ^c^	−29.94 ± 1.80 ^a^	0.27 ± 0.01 ^a^
L2	164.70 ± 0.95 ^a^	−7.78 ± 8.07 ^b^	0.3 ± 0.03 ^b^
L3	91.22 ± 0.86 ^b^	−29.30 ± 4.39 ^a^	0.26 ± 0.01 ^a^
L4	64.89 ± 0.90 ^d^	−29.70 ± 1.56 ^a^	0.27 ± 0.01 ^a^
L5	63.53 ± 1.13 ^d^	−29.33 ± 1.76 ^a^	0.27 ± 0.01 ^a^

**Table 3:** LE4 and LE5 storage stability data under 5 ± 3 °C conditions. The LE4 formulation contained OE at 90 mg, Lipoid S100 at 45 mg and Lipoid S75 at 45 mg. The LE5 formulation contained OE at 60 mg, Lipoid S100 at 60 mg and Lipoid S75 at 60 mg.

	Formulation Codes			
	LE4		LE5	
	Initial	1 mon.	Initial	1 mon.
Mean Particle Size (nm)	127.90 ± 1.81	129.70 ± 1.22	130.90 ± 2.13	132.20 ± 2.60
Zeta Potential (mV)	−0.31 ± 0.13	0.22 ± 0.26	−7.81 ± 0.21	−21.80 ± 1.31
PDI	0.30 ± 0.01	0.30 ± 0.03	0.30 ± 0.02	0.30 ± 0.02
Encapsulation Efficiency of CA (%)	96.99 ± 1.21	94.56 ± 1.53	97.34 ± 1.22	95.78 ± 1.18

**Table 4:** The composition of liposomes: weight ratio, mass and volume information and formulation codes.

Formulation Code	Composition		Ratio
	Lipoid S75	Lipoid S100	
L1	300	-	-
L2	-	300	-
L3	150	150	1:1
L4	100	200	1:2
L5	75	225	1:3

**Table 5:** The composition of OE loaded liposome formulations.

Formulation Code	Code	Composition of the Formulations (mg)			Ratio
		OE	Lipoid S100	Lipoid S75	
L2	LE1	90	90	-	1:1
	LE2	60	120	-	1:2
	LE3	30	150	-	1:5
L3	LE4	90	45	45	1:1
	LE5	60	60	60	1:2
	LE6	30	75	75	1:5

## Data Availability

The data presented in this study are available on request from the corresponding author.
